# Pictorial Review of MRI Findings of Glycogen Storage Disease from Children to Young Adults

**DOI:** 10.3390/children12030295

**Published:** 2025-02-27

**Authors:** Yasuo Amano, Mika Ishige, Maki Amano, Naoki Shinoda, Chisato Ando, Ryo Takagi

**Affiliations:** 1 Department of Radiology, Nihon University Hospital, 1-6 Kanda-Surugadai, Tokyo 101-8309, Japan; 2 Department of Pediatrics, Nihon University Hospital, 1-6 Kanda-Surugadai, Tokyo 101-8309, Japan; 3 Division of Radiological Technology, Nihon University Hospital, 1-6 Kanda-Surugadai, Tokyo 101-8309, Japan

**Keywords:** glycogen storage disease, magnetic resonance imaging, children, hepatomegaly, hepatic tumors

## Abstract

Glycogen storage diseases (GSDs) are rare, inherited disorders of glycogen metabolism caused by a deficiency of enzymes or transporters. GSDs involve the liver, kidneys, skeletal muscles, and heart of children and young adults. The complications involving these organs affect the prognosis of patients with GSDs. Magnetic resonance imaging (MRI) is useful for identifying the complications of GSDs and monitoring the response to treatments owing to its ability of tissue characterization and the lack of a need for ionizing radiation. This pictorial review describes the MRI sequences used for GSDs, presents clinical examples, and emphasizes the pivotal role of MRI as an imaging tool in diagnosing complications associated with GSDs. MRI should be performed at least every year in patients with GSDs and hepatic tumors or myocardial scarring. Further MRI sequences that can be used to quantify the severity of GSDs are discussed.

## 1. Introduction

**Figure 1:**
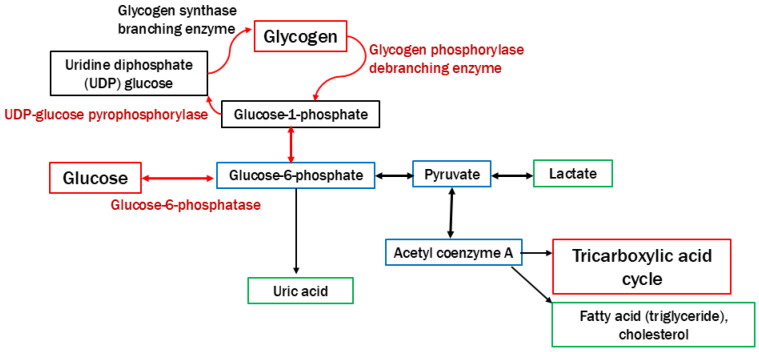
Pathway of glycogen synthesis and degradation. The roles of glycogen are the disposal of excess glucose and the provision of energy during fasting or physical activity. The deficiency of enzymes and transporters leads to the abnormal accumulation of glycogen in multiple organs.

Glycogen storage diseases (GSDs) are rare, inherited disorders of glycogen metabolism caused by a deficiency of enzymes or transporters [1,2,3,4]. The incidence of GSDs is approximately 1 case per 20,000–40,000 live births. Most types of GSDs are inherited in an autosomal recessive manner, and GSD type IX is inherited in an X-linked manner. The roles of glycogen are the disposal of excess glucose and the provision of energy during fasting or physical activity (Figure 1). Therefore, hypoglycemia during fasting is initially observed in pediatric patients with GSDs [1,2,3,4,5,6]. Because glycogen is stored in the liver, skeletal muscles, and kidneys, these organs are affected by the excessive accumulation of glycogen or similar substrates (e.g., dextrin) in patients with GSDs [1,2,3,4,6]. Dietary therapies involving the continuous uptake of glucose and uncooked cornstarch can prevent hypoglycemia associated with GSDs, which would help patients to avoid neurological complications, growth retardation, and hepatic and renal complications [1,3,4,5,6]. Nonetheless, liver cirrhosis, hepatic tumors, neutropenia, renal impairment, and myocardial disorders may contribute to the prognosis of GSDs in children and young adults [1,2,3,4,7,8,9,10,11,12,13]. Liver or kidney transplantation or various gene therapies may be performed in patients with GSDs associated with critical complications [1,4,6,14]. Tumor resection is performed in patients with GSDs and hepatocellular carcinoma [10]. Table 1 summarizes the types of GSDs presented in this review.

Diagnostic imaging tools are useful for identifying and monitoring the complications of GSDs. Ultrasonography is the first-line method for observing the complications in pediatric patients with GSDs owing to its accessibility, functional assessment capabilities, and lack of ionizing radiation [3,6,12]. Magnetic resonance imaging (MRI) has advantages over ultrasonography in high soft tissue contrast, including adipose tissue [3,8,9,10,11,12,13]. The use of contrast agents, including nonspecific gadolinium-based agents (e.g., gadoterate meglumine) and hepatocyte-specific agents (e.g., gadoxetate disodium: Gd-EOB-DTPA), allows for clear recognition, characterization, and monitoring of the complications of GSDs [8,9,10,11,12,13].

The purpose of the pictorial review is to present body MRI sequences that are available for identifying and monitoring the complications of GSDs and as clinical examples. We discuss the future directions for MRI in GSDs, such as the possible application of quantitative MRI.

## 2. MRI Sequences and Practical Performance

**Figure 2:**
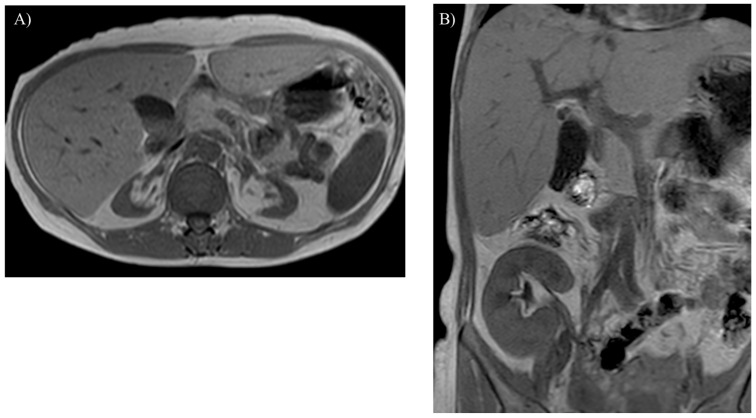
Renal atrophy is observed on T1-weighted imaging in a 40-year-old woman with glycogen storage disease type Ia. The atrophic kidneys lose corticomedullary contrast on T1-weighted imaging (**A**). A transplanted kidney shows normal corticomedullary contrast on T1-weighted imaging (**B**).

**Figure 3:**
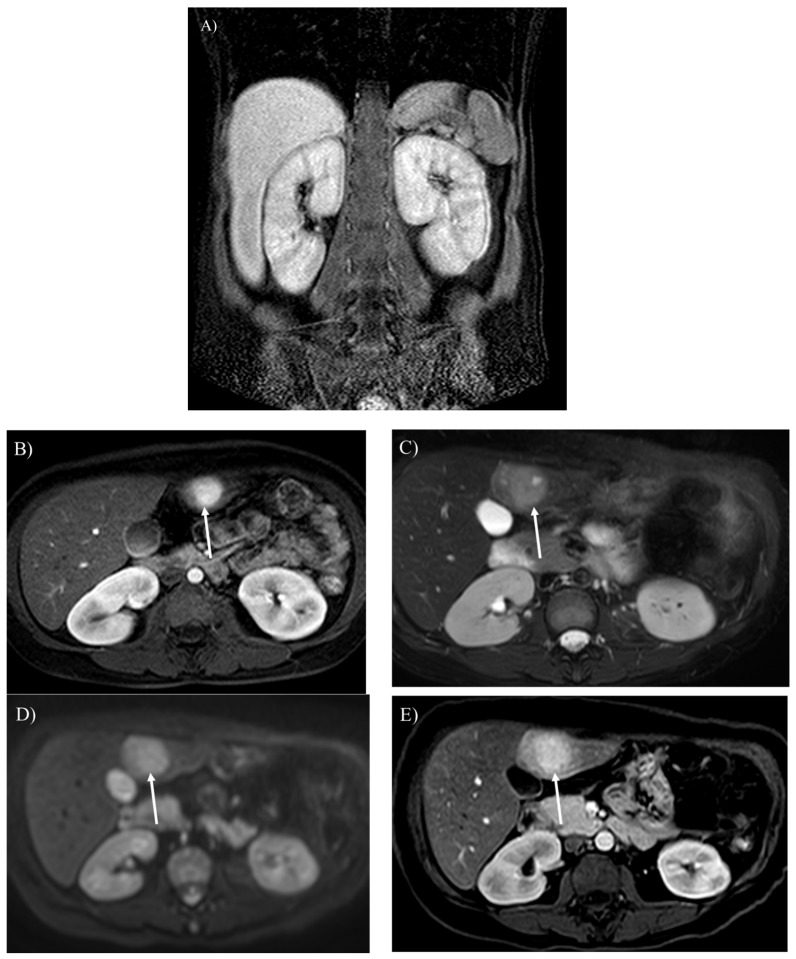
A 20-year-old woman with glycogen storage disease type Ia exhibiting enlargement of the kidney (**A**). Dynamic MRI (**B**) reveals a hepatic adenoma presenting strong enhancement in the arterial phase (arrow). The hepatic adenoma shows high intensity on T2-weighted (**C**) and diffusion-weighted imaging (**D**), (arrows). The tumor gradually enlarged over 13 years (**E**), (arrow).

**Figure 4:**
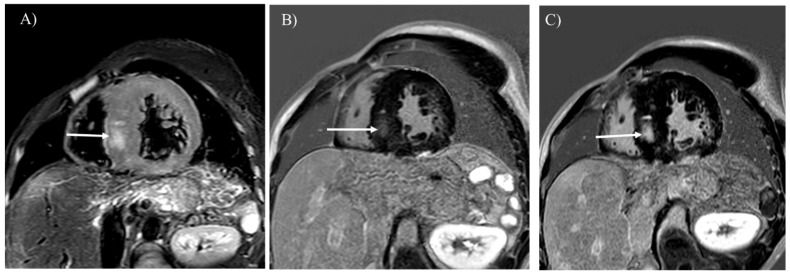
T2-weighted imaging (**A**) showed myocardial hypertrophy and edema in a 27-year-old man with glycogen storage disease type IIIa (arrow). He had undergone liver transplantation 8 years before. Late gadolinium enhancement (LGE) imaging (**B**) identifies a myocardial scar in the hypertrophied septum (arrow). The location of LGE is identical to that of myocardial edema (**A**,**B**); (arrows). Progression of the myocardial scarring was observed at 30 years (**C**), (arrow).

**Figure 5:**
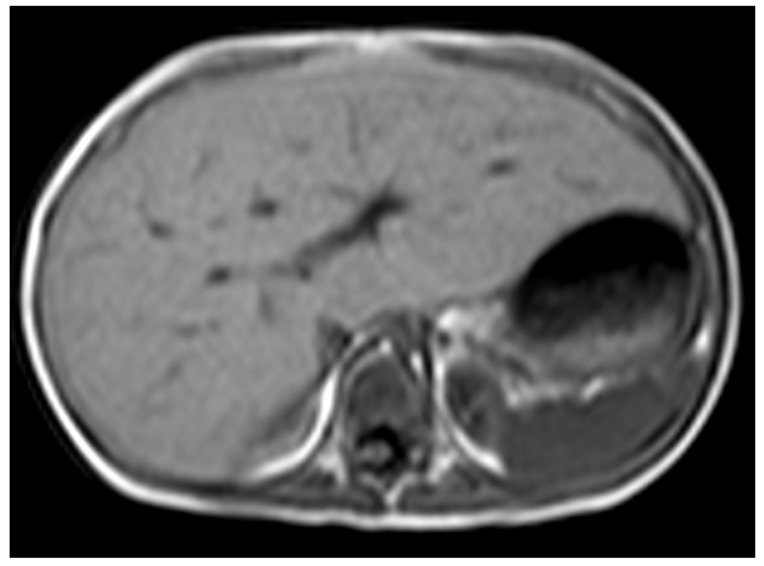
A 3-year-old boy with glycogen storage disease type IXa presents hepatomegaly but not splenomegaly. This type is known to improve with aging.

**Figure 6:**
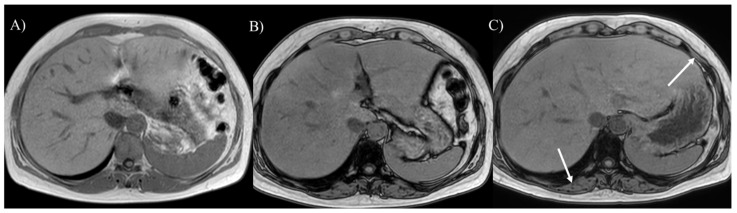
A 16-year-old boy with glycogen storage disease type Ib. Compared to in-phase T1-weighted imaging (**A**), opposed-phase T1-weighted imaging (**B**) reveals a signal drop in the liver, suggesting hepatic steatosis. After 7 years, the liver signal almost normalizes, whereas the spinal erector and intercostal muscles do not grow fully (**C**), (arrows).

**Figure 7:**
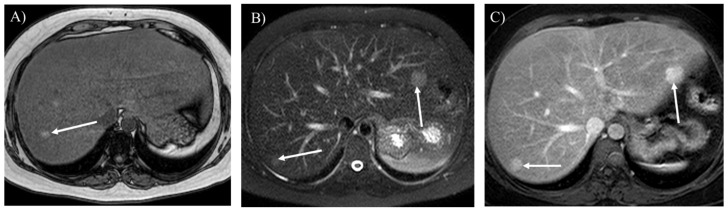
Opposed-phase T1-weighted imaging (**A**) demonstrates hepatic steatosis and hepatic adenoma in a 31-year-old woman with glycogen storage disease type Ia (arrow). T2-weighted (**B**) and contrast-enhanced T1-weighted (**C**) imaging show hepatic adenomatosis (arrows).

**Figure 8:**
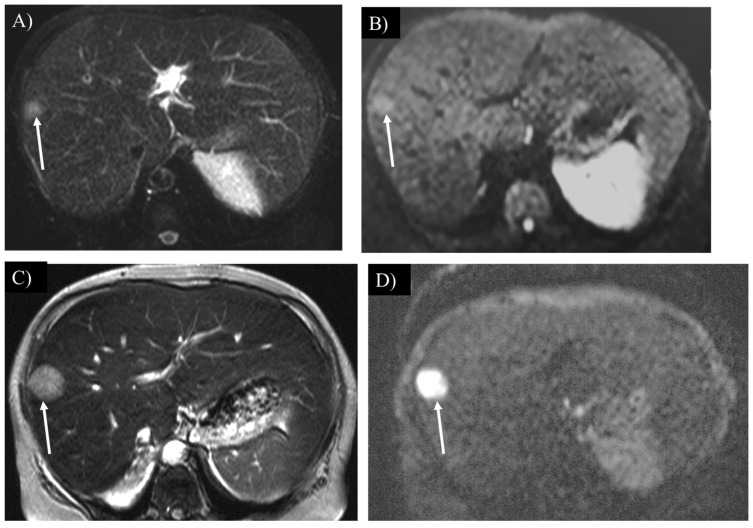
A liver tumor shows high intensity on T2-weighted (**A**) and diffusion-weighted imaging (**B**) in a 55-year-old woman with glycogen storage disease type Ia (arrow). The tumor grew rapidly over 1 year (**C**), (arrow). The tumor shows marked hyperintensity on diffusion-weighted imaging (**D**), (arrow), which is pathologically proven to be hepatocellular carcinoma.

MRI reveals an enlargement or atrophy of various organs in the body (Figure 2A, Figure 3A,B, Figure 4A and Figure 5). In-phase and opposed-phase T1-weighted imaging, which is dual-echo T1-weighted imaging, reveal hepatic steatosis and its severity (Figure 6 and Figure 7A) [10]. T1-weighted imaging is also useful for detecting fatty infiltration in skeletal muscles and visualizing the corticomedullary contrast of the kidneys (Figure 2) [15,16]. T2-weighted imaging is useful for visualizing tissue edema (Figure 4A) [17]. Both T2-weighted and diffusion-weighted imaging visualize hepatic tumors (Figure 3C,D, Figure 7B and Figure 8) [8]. Dynamic gadolinium-enhanced MRI visualizes and diagnoses hepatic tumors with high contrast resolution (Figure 3B,E and Figure 7C) [7,8,9,10]. Cine steady-state free precession imaging provides the structures and functional parameters of the heart with high reproducibility [12]. Late gadolinium enhancement (LGE) imaging visualizes myocardial scarring in the hypertrophied myocardium associated with GSD type IIIa (Figure 4B) [11,12,13]. Table 2 summarizes the body MRI sequences available for evaluating the various complications of GSDs. The MRI protocols of the abdomen and heart in patients with GSDs are identical to those without GSDs [7,8,9,10,11,12,18].

## 3. MRI Findings of Complications Associated with GSDs

### 3.1. Hepatic Involvement

Hepatomegaly is observed in most patients with GSD type I, which occurs in young children because of the early accumulation of glycogen (Figure 5) [1,3,5,6]. Hepatosplenomegaly occurs as hepatic dysfunction progresses [1]. Liver transplantation can be performed in patients with serious liver cirrhosis [1,6]. MRI is useful for monitoring the size and morphology of the liver and spleen and making a diagnosis of liver cirrhosis.

Hepatic steatosis is often associated with GSDs because of malnutrition and lipid metabolism disorders [3,6,7]. Dual-echo T1-weighted imaging reveals hepatic steatosis (Figure 6A,B and Figure 7A) [10]. It is also useful for monitoring the severity of hepatic steatosis. The liver signal normalizes on opposed-phase T1-weighted imaging in patients with GSD type I following successful dietary therapies (Figure 6B,C).

**Figure 9:**
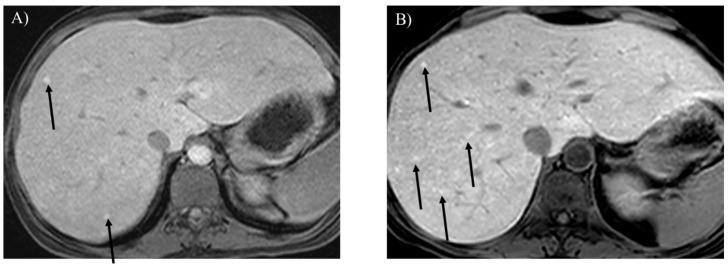
Regenerative nodules show a tiny high signal on the hepatobiliary phase of Gd-EOB-DTPA-enhanced imaging (**A**) in a 24-year-old woman with glycogen storage disease type Ia (arrows). The number of regenerative nodules increased significantly after 14 years as liver dysfunction progressed (**B**), (arrows).

Regenerative nodules are associated with liver cirrhosis. Gd-EOB-DTPA-enhanced imaging is useful for differentiating hepatic nodules, and regenerative nodules show high signal intensity in the hepatobiliary phase (Figure 9) [18]. As the liver parenchymal damage progresses, the number of regenerative nodules increases, as identified by MRI (Figure 9B).

Hepatic adenoma is found in 16–75% of patients with GSD type I [1,2,3,6,7,8,9]. Some genetic alternations or inadequate metabolic control may contribute to a larger hepatic adenoma in patients with GSD type Ia [3]. Different from hepatic adenomas without GSDs, those associated with GSDs are often found in male patients, and there may be multiple tumors (i.e., hepatic adenomatosis) which might increase in size (Figure 3B,E and Figure 7B,C) [6,18,24]. The hepatic adenoma associated with GSDs are not related to contraceptives. It is clinically problematic to differentiate between a growing hepatic adenoma and hepatocellular carcinoma. Indeed, hepatic adenoma can transform into hepatocellular carcinoma in the older patients with GSDs, exhibiting similar imaging appearances in GSDs (Figure 3C–E, Figure 7B and Figure 8) [10,18]. Multimodality imaging surveillance should be performed every 1 or 2 years once a hepatic tumor presenting strong enhancement in the arterial phase is identified in patients with GSDs [6,8,10].

### 3.2. Renal Impairment

Glycogen and similar materials accumulate in the kidneys, which induces renal complications in patients with GSDs [1,2,3,5,6,15]. MRI is insensitive to renal calculi associated with GSDs, whereas it can detect an enlargement or atrophy of the kidneys (Figure 2A and Figure 3A). Nephromegaly is found in a majority of patients with GSD type I, while renal dysfunction and atrophy progress in approximately half of patients, especially under inadequate metabolic control [25]. T1-weighted imaging identifies renal impairment as decreased corticomedullary contrast in the kidneys (Figure 2A) [16]. MRI can also be used to investigate transplanted kidneys without contrast agents (Figure 2B).

### 3.3. Skeletal Muscular Growth Retardation

Glycogen is consumed in the skeletal muscles during physical activity. Growth retardation, atrophy, and clinical symptoms, including muscular weakness and cramping, are observed in children and young adults with GSDs [1,2]. MRI may be useful for evaluating volume and fatty infiltration in the skeletal muscles (Figure 6B). Because MRI can visualize the distribution of the muscles involved in a wide range of views, it may be useful for distinguishing GSDs from muscular dystrophies and neuromuscular disorders [26].

### 3.4. Myocardial Involvement

GSD type IIIa is known to involve the myocardium in approximately half of patients and can lead to sudden cardiac death because of severe myocardial hypertrophy or glycogen accumulation in the conduction system [1,2,4,27]. MRI is the best imaging tool for evaluating tissue characterization within the myocardium. Myocardial edema and scars associated with GSD type IIIa are visualized with T2-weighted and LGE imaging, respectively (Figure 4) [11,12,13,14,17]. LGE is useful for observing myocardial scarring that can progress even after liver transplantation (Figure 4B,C). The differential diagnoses of myocardial involvement in GSDs include hypertrophic cardiomyopathy, Anderson–Fabry disease, Noonan syndrome, and mitochondrial cardiomyopathies, which exhibit myocardial hypertrophy in children and young adults [12,19,28]. Given that myocardial scarring identified by LGE is predictive of the prognosis of GSD type III, similarly to hypertrophic cardiomyopathy, MRI should be performed every year to evaluate cardiac function and myocardial scarring [19].

## 4. Discussion

### 4.1. Advantages and Limitations of MRI

Both ultrasonography and MRI do not require ionizing radiation, which is a large advantage over CT and nuclear medicine examination in children, adolescents and patients who need long-term follow-up. Ultrasonography is the first imaging technique to evaluate complications of GSDs owing to its lower cost, good accessibility, ability to measure cardiac function, and its rich clinical experience [3,4,13,20,21]. MRI is recommended in addition to ultrasonography, especially in patients older than 10 years, because MRI has several advantages, including high tissue contrast, operator independence, capability for whole body scanning, and tissue quantification [3,11,12,16,18,22,26]. For instance, diffusion-weighted imaging and dynamic Gd-EOB-DTPA imaging, both of which use fat-suppression, clearly identify hepatic tumors in GSD patients with concomitant hepatic steatosis (Figure 7 and Figure 8) [8,10,18]. Because of large echogenicity of fatty tissues, severe steatosis interrupts the characterization of hepatic tumors by ultrasonography [20]. Renal parenchymal tissues can be easily evaluated by T1-weighted imaging (Figure 2A) [15,16]. LGE visualizes myocardial scarring associated with GSD type IIIa (Figure 3B) [11,12,13]. Fat fraction in the liver is quantified by Dixon sequence [22]. The limitations of MRI are its high cost, need of sedation and use of contrast agents in some pediatric patients with GSDs.

### 4.2. Clinical Relevance of MRI

Complications predict the worse prognosis of GSDs. MRI identifies malignant transformation of hepatic adenoma to hepatocellular carcinoma that should be resected (Figure 8) [10]. MRI also supports monitoring of effects of dietary therapies by exhibiting liver cirrhosis associated with regenerative nodules, renal atrophy, and myocardial hypertrophy (Figure 2A and Figure 9). If myocardial scarring is related to critical arrhythmia or heart failure as in the cases of hypertrophic cardiomyopathy, LGE can indicate Holter electrogram and implantable cardioverter defibrillator installation in patients with GSD and myocardial scarring (Figure 4B,C) [29]. MRI should be regularly performed to investigate hepatic tumors or myocardial scarring in pediatric patients with GSDs [3].

## 5. Future Directions

MRI is able to observe the whole body with high spatial and contrast resolution, a wide range of views, and no need for radiation. Tobaly et al. [26] applied whole-body MRI to investigate the distribution and severity of the skeletal muscles involved by GSD type III.

Some quantitative MRI techniques can be applied to GSDs to evaluate their severity. Chemical shift exchange sequences and ^13^C MR spectroscopy can be used to estimate glycogen in the organs [20,21]. The Dixon sequence can be used to quantify hepatic steatosis associated with GSDs, because it is an established technique for measuring fat fraction in the liver [3,22]. Quantitative T1 mapping is used to evaluate myocardial fibrosis and edema in patients with GSD IIIa [13,29]. Because T1 mapping does not require gadolinium contrast agents for myocardial tissue characterization, it may be useful for patients with GSD and renal impairment. T1 mapping may also be used to evaluate the fat fraction or degree of fibrosis in the liver [23].

## 6. Conclusions

GSDs, which are rare, inherited metabolic disorders of glycogen metabolism, involve multiple organs from childhood to young adulthood. In this pictorial essay, we reviewed the MRI features of their complications to guide and monitor the appropriate treatments for GSDs. Because some of these complications indicate or predict an unfavorable prognosis in pediatric patients with GSDs, MRI should be regularly performed to investigate hepatic tumors or myocardial scarring.

## Figures and Tables

**Table 1:** Types of glycogen storage diseases presented in this review.

Type	Clinical Findings
**Ia**	Deficiency of glucose-phosphatase, AR
	HA or HCC with hepatomegaly, hepatic steatosis,
	renal impairment, anemia and osteopenia
**Ib**	Deficiency of glucose-phosphatase transporter, AR
	Hepatomegaly, neurtropenia, Crohn-like bowel disease,
	myasthenia gravis, thyroid dysfunction
**IIIa**	Deficiency of glycogen debrancher enzyme, AR
	Liver cirrhosis, HA or HCC, myopathy, cardiomyopathy
**IXa**	Deficiency of phosophorylase kinase, X-linked (most common)
	Hepatomegaly and steatosis (improve with aging)

Hepatic involvement is common to all types of glycogen storage diseases, while there are some differences in clinical features between the types. AR: autosomal recessive inheritance, HA: hepatic adenoma, HCC: hepatocellular carcinoma, X-linked: X-linked inheritance.

**Table 2:** Body MRI sequences used for evaluating complications of glycogen storage diseases.

Sequences	MRI Findings
All	Organomegaly, renal atrophy
T1-weighted imaging [16]	Perfusion, hepatocytes, renal parenchyma
Dual echo (in/opposed phase) [7]	Fatty infiltration
T2-weighted imaging [17]	Tumors, edema
Cine imaging [12,19]	Cardiac morphology and function
Late gadolinium enhancement [11]	Myocardial scar
Chemical shift exchange [20]	Glycogen quantification
^13^C spectroscopy [21]	
Dixon [22]	Fat fraction quantification
T1 mapping [13,23]	Fat or fibrosis quantification

Parentheses represent the references cited in this article. MRI with multiple imaging sequences is useful for identifying and monitoring complications of glycogen storage diseases. Imaging protocols for glycogen storage diseases are identical to those used in the clinical routine. Quantitative sequences will be used for accurate and continuous estimation of severity of glycogen storage diseases.

## Data Availability

Data availability is not applicable to this article as no new data were created or analyzed in this study.
